# Epidemiology of khat (*Catha edulis*) consumption among university students: a meta-analysis

**DOI:** 10.1186/s12889-019-6495-9

**Published:** 2019-02-04

**Authors:** Getinet Ayano, Kalkidan Yohannis, Mebratu Abraha

**Affiliations:** 1 Research and Training Department, Amanuel Mental Specialized Hospital, POBOX: 1971, Addis Ababa, Ethiopia; 2 Department of Psychiatry, Dilla University, Dilla, Ethiopia; 3 Department of Psychiatry, Paulo’s millennium medical college, Addis Ababa, Ethiopia

**Keywords:** Epidemiology, Khat use, University students, Meta-analysis

## Abstract

**Background:**

Khat is amphetamine-like substance commonly consumed by students when they wish to study for long hours especially during examination periods. Khat consumption is associated with increased rates of cardiovascular problems, stroke, myocardial infarction, cardiomyopathy, periodontal disease, gastritis, poor oral hygiene, psychosis, decreased quality of life, and poor academic performance.

**Methods:**

PubMed, EMBASE, and SCOPUS were systematically searched without restriction by publication status. Publications were screened according to predefined inclusion criteria. Twenty-five articles were included. Random effect model was used to calculate weighted prevalence, odds ratio (OR) and corresponding 95% confidence interval (CI). We assessed the risk of publication bias by using funnel plot and Eggers’s regression tests.

**Results:**

The pooled prevalence of current khat use among university students was 14.16% (95% CI; 11.87–16.81). The pooled prevalence of current khat use was highest in Saudi Arabia 18.85% and it was 13.59% in Ethiopia and 13.04% in Yemen. In addition, the current pooled prevalence of khat use was higher in men at 19.26% than in women 6.41%. Regarding lifetime khat use, in this study, the pooled prevalence of lifetime khat use was 27.31%. The lifetime prevalence was higher in men at 31.47% than in women 11.79%. Moreover, the lifetime prevalence of khat use was 43.27% in Yemen, 37.32% in Saudi Arabia, and 24.82% in Ethiopia. We found significantly increased odds of current (OR 3.59; 95%CI 2.01–6.41) as well as lifetime (OR 3.48; 95%CI 2.09–5.78) khat chewing in men as compared to women.

**Conclusion:**

The pooled prevalence of current and lifetime khat consumption was 14.16 and 27.31%, respectively. Both the current and lifetime prevalence of khat use was higher in men than in women. In addition, both the current and lifetime prevalence estimates of khat consumption were low in Ethiopia than in Saudi Arabia. Moreover, the odds of both current and lifetime khat consumption were higher in male students than in female students. Programmes that specifically aim at increasing awareness and that most motivate reduced khat consumption among university students were recommended**.**

**Electronic supplementary material:**

The online version of this article (10.1186/s12889-019-6495-9) contains supplementary material, which is available to authorized users.

## Background

Khat (*Catha edulis*) is “amphetamine-like” substance or stimulant which is consumed widely in some parts of Africa as well as Arabian Peninsula and Diaspora communities from these countries [1, 2]. In some countries located in Africa and the Arabian Peninsula, chewing the leaves of khat is socioculturally embedded and commonly practiced mainly due to its stimulating property [2, 3].

For those people living in some societies in Yemen and other east African countries chewing the leaves of khat is acceptable and habitual practice particularly among Muslims [4, 5]. The fresh leaves of khat are chewed daily on regular basis mainly in the afternoon, although starting in the morning is seen in some people. In addition, chewing khat is more popular in some social gatherings such as funerals and wedding election time and wedding parties.

Epidemiological evidence showed that khat consumption is associated with an increased rates of cardiovascular problems [6–8], stroke [9–11], myocardial infarction and cardiomyopathy [12, 13], periodontal disease, stomatitis, esophagitis and gastritis [14, 15], periodontitis, gingivitis and poor oral hygiene [16–19], psychosis [20, 21], decreased quality of life [22], and poor academic performance [23, 24].

Khat consumption is also a common practice among university students [6]. University students commonly chew khat when they want to study for long hours mostly during examination periods [25]. According to scientific evidence, there is a recent increase in the consumption of khat among university students. For example, in one study conducted among male students of Aden University, the prevalence of khat chewing increased from 35 to 90% in 5 years [26]. In other study conducted in Saudi Arabia among students of age 15 to 25 years, the prevalence of khat consumption was found to be 37.7% among male students compared to only 3.8% among females [27]. In one of Ethiopian study conducted in Addis Ababa, 15.9% of male students regularly chew khat [6].

In view of the above issues, we undertook a meta-analysis of published observational studies conducted among university students on khat use to systematically investigate: [1] the prevalence of current and lifetime hat use, [2] the existing differences in the risk of khat consumptions between men and women, and to formulate recommendations for future research.

## Methods

A systematic literature search was conducted following the “Preferred Reporting Items for Systematic Reviews and Meta-Analyses (PRISMA)” guidelines [28]. We consulted three databases (EMBASE, PubMed, and Scopus) for our search. We searched PubMed using the following terms and keywords ((khat OR *Catha edulis* OR khat use OR khat consumptions OR khat chewing OR khat abuse OR khat dependence OR khat use disorder OR substance OR substance use OR psychoactive substance OR substance use disorder OR substance abuse)) AND (student OR university student OR college student). For the remaining two databases (EMBASE and Scopus) we used specific subject heading related to the above keywords employed in PubMed. The citations retrieved from each database were saved in our endnote library at the moment of searching. After completing the search for each database, we imported the completed citations from the three databases into a unique EndNote library and saved after excluding duplicates for this research. We also scanned the reference lists of eligible studies to identify additional studies of relevance to this review. In addition, we looked manually to discover possible relevant published as well as grey literature.

### Eligibility criteria

Studies meeting the following criteria were included in this meta-analysis: First, the observational study in design type (including cross-sectional and case-control study design); second, the outcome of interest was either khat use (current and lifetime use) or khat dependence among university students; third reported the magnitude of khat use and dependence and determined the risk khat use for male and female university students. Moreover, we excluded commentaries, reviews, editorials, and studies not published in the English language. Two investigators (GA and MA) screened the relevance of the studies based on their title and abstract before the retrieval of full-text articles. Then the full-text articles were further evaluated them for their eligibility independently by two investigators (GA and MA). We resolved disagreements by discussing with the third investigator (KY).

### Methods for data extraction

We utilized a standard data extraction form to identify relevant data from full-text studies included in our meta-analysis. Two investigators (GA and MA) independently extracted the data. We extracted the following information: first author’s last name, country, a sample size of the participants, year of publication, study setting (community, clinical or institution), instrument used for measuring outcome, and the prevalence of khat use and dependence.

### Methods for data analysis and quality assessment

We used a random-effect model to calculate the pooled prevalence, odds ratios and 95% CIs in our meta-analysis [29]. Comprehensive meta-analysis software version3 was used for analysis. We used Cochran Q and the I^2^statistics to evaluate the presence of heterogeneity [29]. The magnitude of *I*^2^ statistic values such as 25, 50 and 75% was used to show low, medium and high heterogeneity, respectively [30]. We evaluated the quality of the studies included in our meta-analysis using the Newcastle-Ottawa scale (NOS) with modifications [31]. The main domains of the NOS include the representativeness of the sample size of the participants, the measurements of khat use, comparisons between the participants and quality of statistical analysis. During the quality assessment, the agreement between the two investigators (GA and MA) was evaluated by using agreement beyond chance (unweighted Kappa). The values 0, 0.01–0.20, 0.21–0.40, 0.41–0.60, 0.61–0.80, and 0.81–1.00 were used to represent poor, slight, fair, moderate, substantial, and almost perfect agreements, respectively [32].

We assessed the risk of publication bias by using funnel plot and Eggers’s regression tests.

## Results

### Study selection

**Fig. 1:**
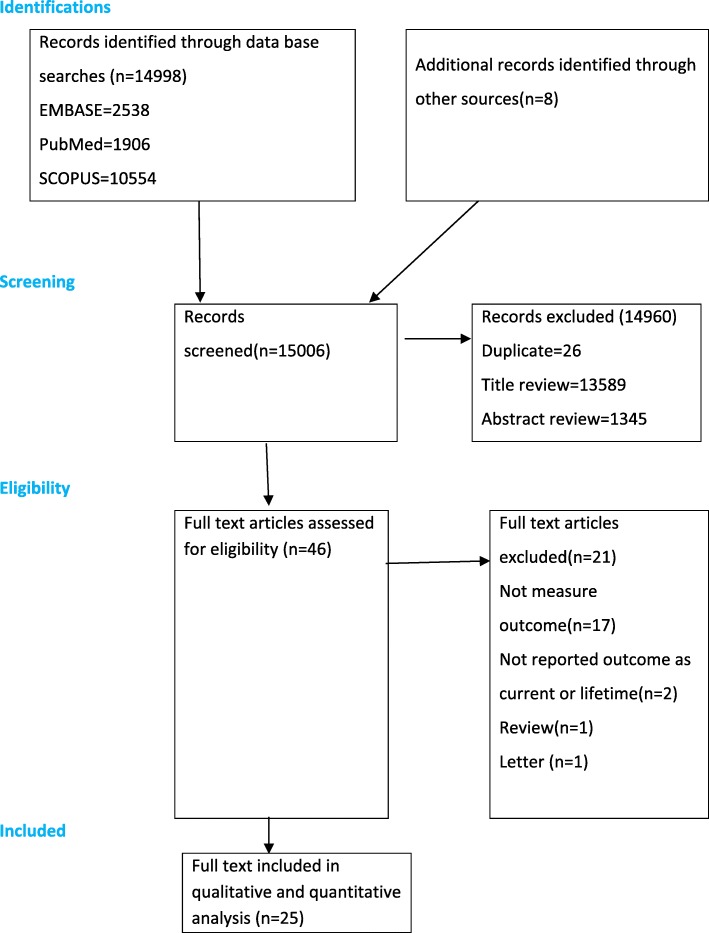
PRISMA flowchart of review search

The electronic search identified 14,998 articles. Additionally, we identified eight citations through a manual search of the reference lists of the remaining articles. A total of 14,960 were excluded based on the review of the abstract, titles and duplicate as they did not meet the inclusion criteria (Fig. 1). Then, the full text of 46 articles was retrieved for further screening and 21 of these were excluded. Finally, 25 articles that qualified the inclusion criteria were included in this study.

### Study characteristics

The characteristics of studies included in this meta-analysis were indicated in Table 1. In this meta-analysis, a total of 24,136 participants from 25 studies were included in the analysis. Selected studies were conducted between 2002 and 2017. All studies included in our analysis used a cross-sectional study design to determine khat use among university students. Most of the studies reported response rate. Most of the studies were conducted in Ethiopia (*n* = 19) [25, 33–50] and the others were conducted in Saudi Arabia (*n* = 3) [27, 51, 52], Yemen (n = 3) [53–55] (See Table 1). Of the included studies, 18 of the studies assessed current khat use and 21 of the studies determined lifetime khat use.

**Table 1:** Characteristics of included studies

Author(year) (reference number)	Sample size	Response rate	Country	Type of use	Outcome (magnitude current and lifetime khat use)
Dida N. et.al (2014) [33]	603	97.9	Ethiopia	Current use	Overall 16.92%(n/*N* = 102/603)
Tesfaye G,et al. (2013) [34]	1022	98.3%	Ethiopia	Current use	Overall 23.6%(n/*N* = 241/1022) Men 28.7%(n/*N* = 223/777) Women 7.3% n/*N* = 18/245)
				Lifetime	Overall 41%(n/*N* = 419/1022) Men 47.6%(n/*N* = 370/777) Women 20% (n/*N* = 49/245)
Hagos EG, et. Al (2013) [35]	271	100%	Ethiopia	Ever use	Overall 9.2%(n/*N* = 25/271)
				Current use	Overall 4%(n/N = 11/271)
Mekonnen T. et.al (2017) [36]	725	97.05%	Ethiopia	Current use	Overall 10.20% (n/*N* = 74/725) Men 11.83%(n/*N* = 57/482) Women 7%(n/*N* = 17/243)
Tadesse M. et.al (2014) [37]	611	98%	Ethiopia	Life time use	Overall 41.08% (n/*N* = 251/611) Men 44.9 (n/*N* = 195/434) Women 31.6% (n/*N* = 56/177)
Eshetu E.et al. (2006) [38]	564	89%	Ethiopia	Current use	Overall 14.36% (n/*N* = 81/564) Men 17.9(n/*N* = 77/431) Women 3%(n/N = 4/133)
				lifetime use	Overall 31.91% (n/*N* = 180/564) Men 37.2(n/*N* = 160/431) Women 14.9%(n/N = 20/133)
Shiferaw D.et al. (2017) [39]	600	92.6%	Ethiopia	Life time use	Overall 33.3%(n/*N* = 200/600)
Kassa A. et al. (2016) [40]	586	94.5%	Ethiopia	Current use	Overall 16.3%(n/*N* = 98/586) Men 18.58%(n/*N* = 89/479) Women 8.41%(n/N = 9/107)
				Life time use	Overall 24.1%(n/*N* = 141/586) Men 26.93%(n/*N* = 129/479) Women 11.21%(n/N = 12/107)
Gebreslassie M. et.al (2013) [41]	756	98.7%	Ethiopia	Current use	Overall 27.9%(n/*N* = 211/756) Men 35.6%(n/*N* = 158/444) Women 17%(n/*N* = 53/312)
				Lifetime use	Overall 28.7%(n/*N* = 217/756) Men 36.5%(n/*N* = 162/444) Women 17.6%(n/*N* = 55/312)
Deressa W. et.al (2010) [42]	622	78%	Ethiopia	Lifetime use	Overall 14.1%(n/*N* = 88/622) Men 17.1%(n/N = 77/426) Women 5.6%(n/*N* = 11/196)
				Current use	Overall 3.7%(n/*N* = 23/622) Men 4.9(n/N = 21/426) Women 1%(n/N = 2/196)
Dessie Y. et.al (2013) [43]	430	97.3%	Ethiopia	Lifetime use	Overall 40%(n/*N* = 172/430)
Adere A. et.al (2017) [44]	655	89.7%	Ethiopia	Lifetime use	Overall 13%(n/*N* = 85/655) Men 16.3%(n/N = 74/454) Women 5.5%(n/N = 11/201)
				Current use	Overall 10.4(n/*N* = 68/655) Men 13%(n/*N* = 59/454) Women 4.5%(n/N = 9/201)
Mulugeta Y. et.al (2015) [45]	745	98.8%	Ethiopia	Lifetime use	Overall 19.6%(n/*N* = 146/745) Men 27.54%(n/*N* = 92/334) Women 13.14%(n/*N* = 54/411)
				Current use	Overall 12.73%(n/*N* = 96/754) Men 18.56%(n/*N* = 62/334) Women 8.27%(n/*N* = 34/411)
Kebede Y. (2002) [46]	1103	87.7%	Ethiopia	Current use	Overall 17.5%(n/*N* = 193/1103)
				Lifetime use	Overall 26.7%(n/*N* = 294/1103)
Reda AA et.al (2012) [25]	1721	91,1%	Ethiopia	Lifetime use	Overall 24.2%(n/*N* = 427/1721)
Abdeta et al. (2017) [47]	619	95.1%	Ethiopia	Current use	Overall 23.9%(n/*N* = 148/619) Men 26.0%(n/*N* = 125/464) Women 14.8%(n/N = 23/155)
				Lifetime use	Overall 26.3%(n/*N* = 163/619)
Astatkie et al. (2015) [48]	1255	97.3	Ethiopia	Current use	Overall 11.1%(n/*N* = 139/1255)
				Lifetime use	Overall 22.8%(n/*N* = 286/1255)
Gebrehanna et al. (2014) [49]	3001	77.5%	Ethiopia	Current use	Overall 12.7%(n/*N* = 380/3001) Men 14.9%(n/*N* = 348/2328) Women 4.8%(n/*N* = 32/673)
				Lifetime use	Overall 24%(n/*N* = 720/3001) Men 27.3%(n/*N* = 636/2328) Women 12.5%(n/*N* = 84/673)
Alsanosy et al. (2013) [51]	3764	91.80	Saudi Arabia	Current	Overall 23.1%(n/*N* = 868/3764) Men 38.5%(n/*N* = 834/2165) Women 2.1%(n/N = 34/1599)
				Lifetime	Overall 24.8%(n/*N* = 935/3764) Men 40.5%(n/*N* = 876/2165) Women 3.7%(n/N = 59/1599)
Quadri et al. (2015) [55]	476	95%	Saudi Arabia	Lifetime	Overall 51.89%(n/*N* = 247/476)
Ageely (2009) [27]	2466	–	Saudi Arabia	Current use	Overall 15.2%(n/*N* = 375/2466)
Kubas Ma et.al. 2015 [53]	460	–	Yemen	Current use	13.04% (n/*N* = 60/460)
Alkhadernl et.al (2009) [54]	100	–	Yemen	Lifetime use	54%(n/N = 54/100)
Dhaifullahet.al (2013) [55]	360	–	Yemen	Lifetime use	33.9%(n/*N* = 122/360)
Dachew BA.et al. (2014) [50]	836	95.8%	Ethiopia	Current use	Overall 13.6%(n/*N* = 114/836) Men 14.7%(n/*N* = 79/538) Women 11.7%(n/*N* = 35/298)
				Lifetime use	Overall 17.9%(n/*N* = 150/836)

### Quality of included studies

We utilized the Newcastle-Ottawa scale (NOS) with modifications to assess the quality of the studies included in our final analysis. Based on our evaluation all 25 studies were of good methodological quality. The investigators agreed the risk of study selection, outcome measurement, and non-response bias was low. The levels of agreements between the reviewers regarding the levels of bias for studies included in this meta-analysis ranged from moderate to almost perfect (Kappa statistic range 0.50–1 (See Table 2).

**Table 2:** Summary of the quality and agreed level of bias and level of agreement on the methodological qualities of included studies in a meta-analysis based on sampling, outcome, response rate and method of analysis

Study	Overall agreement and precision			Nos quality (score on 0 to 9 scale)
	Percentage of agreement	Kappa value	Level of agreement	
Dida et.al. (2014) [33]	75	0.60	Moderate	7
Tesfaye (2013) [34]	100	1	Almost perfect	8
Hagos et.al. (2013) [35]	75	0.60	Moderate	7
Mekonen et.al. (2017) [36]	100	1	Almost perfect	8
Tadesse et.al. (2014) [37]	100	1	Almost perfect	9
Eshetu et.al. (2006) [38]	100	1	Almost perfect	8
Shiferaw et al. (2017) [39]	75	0.60	Moderate	7
Kassa et.al. (2016) [40]	100	1	Almost perfect	9
Gebreslassie et.al (2013) [41]	100	1	Almost perfect	9
Deressa et.al. (2010) [42]	100	1	Almost perfect	9
Dessie et.al. (2013) [43]	75	0.60	Moderate	7
Adere et.al. (2017) [44]	100	1	Almost perfect	8
Reda AA et.al (2012) [25]	100	1	Almost perfect	8
Mulugeta Y. et.al (2015) [45]	100	1	Almost perfect	8
Kebede (2005) [46]	100	1	Almost perfect	9
Abdeta et al. (2017) [47]	100	1	Almost perfect	8
Astatkie et al. (2015) [48]	100	1	Almost perfect	8
Gebrehanna et al. (2014) [49]	100	1	Almost perfect	8
Alsanosy et al. (2013) [51]	100	1	Almost perfect	8
Quadri et al. (2015) [52]	100	1	Almost perfect	9
Ageely (2009) [27]	100	1	Almost perfect	8
Kubas Ma et.al. 2015 [53]	100	1	Almost perfect	9
Alkhader nl et.al (2009) [54]	75	0.50	Moderate	6
Dhaifullah et.al (2013) [55]	75	0.60	Moderate	7
Dachew BA.et al.(2014) [50]	100	1	Almost perfect	9

### The results of a pooled meta-analysis

#### Prevalence of current khat use

**Fig. 2:**
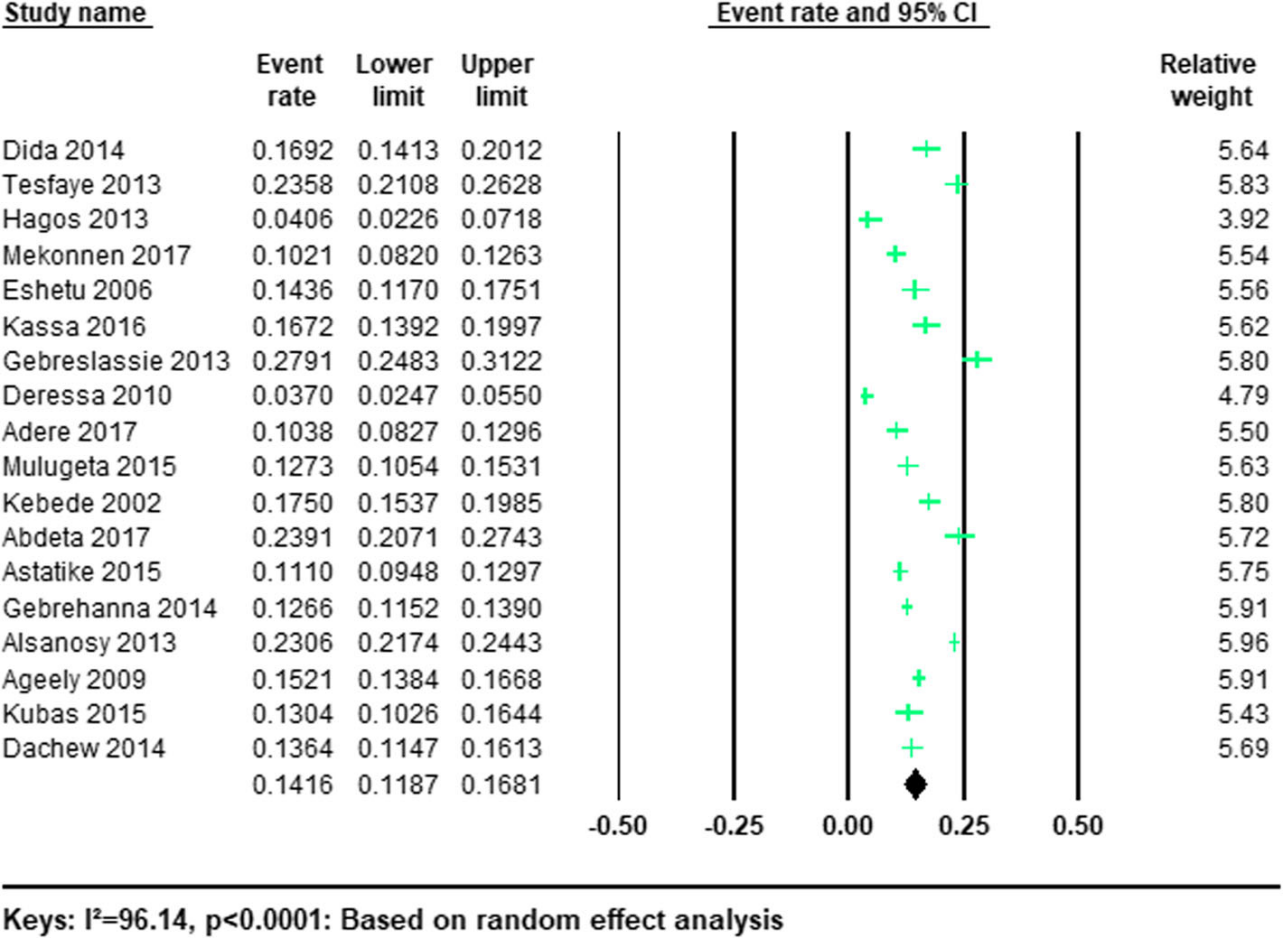
Forest plot of current khat use among university students: a meta-analysis

As demonstrated in Table 1, 18/25 (72%) studies reported the prevalence of current khat use among university students. The total sample size of the participants from the included studies was 19,838. Based on the results of the random-effects method, the pooled prevalence of current khat use was 14.16% (95% CI; 11.87–16.81) and we observed a significant heterogeneity (*I*^2^ = 96.14%; Q = 440.16.13, df = 17, *p* < 0.0001). The forest plots of the prevalence of current khat consumption were shown in Fig. 2.

The pooled prevalence of current khat use was highest in Saudi Arabia (18.85, 95% CI 12.31–27.75) and it was 13.59% (95% CI 11.02–16.62) in Ethiopia and 13.04% (95% CI 10.26–16.44) in Yemen (Table 3). However, in our sensitivity analysis, the difference between the studies was not statistically significant (*P* = 0.312).

**Table 3:** Subgroup analysis of the prevalence of khat use among university students based on random effect analysis

Subgroup	Number of studies	Type of use	Estimates		Heterogeneity		
			Prevalence (%)	95% Confidence interval	I^2^ (%)	Q(df)	*P* value
Sex							
Men	12	Current	19.26	14.05–25.82	98.01	553.56(11)	*P* < 0001
Women	12	Current	6.41	4.12–9.82	91.96	136.78(11)	*P* < 0001
Men	10	Lifetime	31.47	25.73–37.84	96.95	294.79(9)	*P* < 0001
Women	10	Lifetime	11.79	7.53–18.01	95.23	188.52(9)	*P* < 0001
Country							
Ethiopia	15	Current	13.59	11.02–16.62	98.88	1243.10(14)	*P* < 0001
Saudi Arabia	2	Current	18.85	12.31–27.75	98.24	56.82(1)	*P* < 0001
Yemen	1	Current	13.04	10.26–16.44	–	–	–
Ethiopia	17	Lifetime	24.82	21.30–28.72	96.43	447.74(16)	*P* < 0001
Saudi Arabia	2	Lifetime	37.32	15.74–65.48	99.30	141.20(1)	*P* < 0001
Yemen	2	Lifetime	43.27	25.31–63.19	92.37	13.04(1)	*P* < 0001

In our stratified analysis of included studies by gender we found that the current pooled prevalence of khat use was higher in men 19.26%(95%CI 14.05–25.82) than women 6.41%(95%CI 4.12–9.82). A significant heterogeneity was found in both men (*I*^2^ = 98.01; Q = 553.56, df = 11, *p* < 0.001) and women (*I*^2^ = 91.96; Q = 136.78, df = 11, *p* < 0.001). (See Table 3).

#### Prevalence of lifetime khat consumptions

**Fig. 3:**
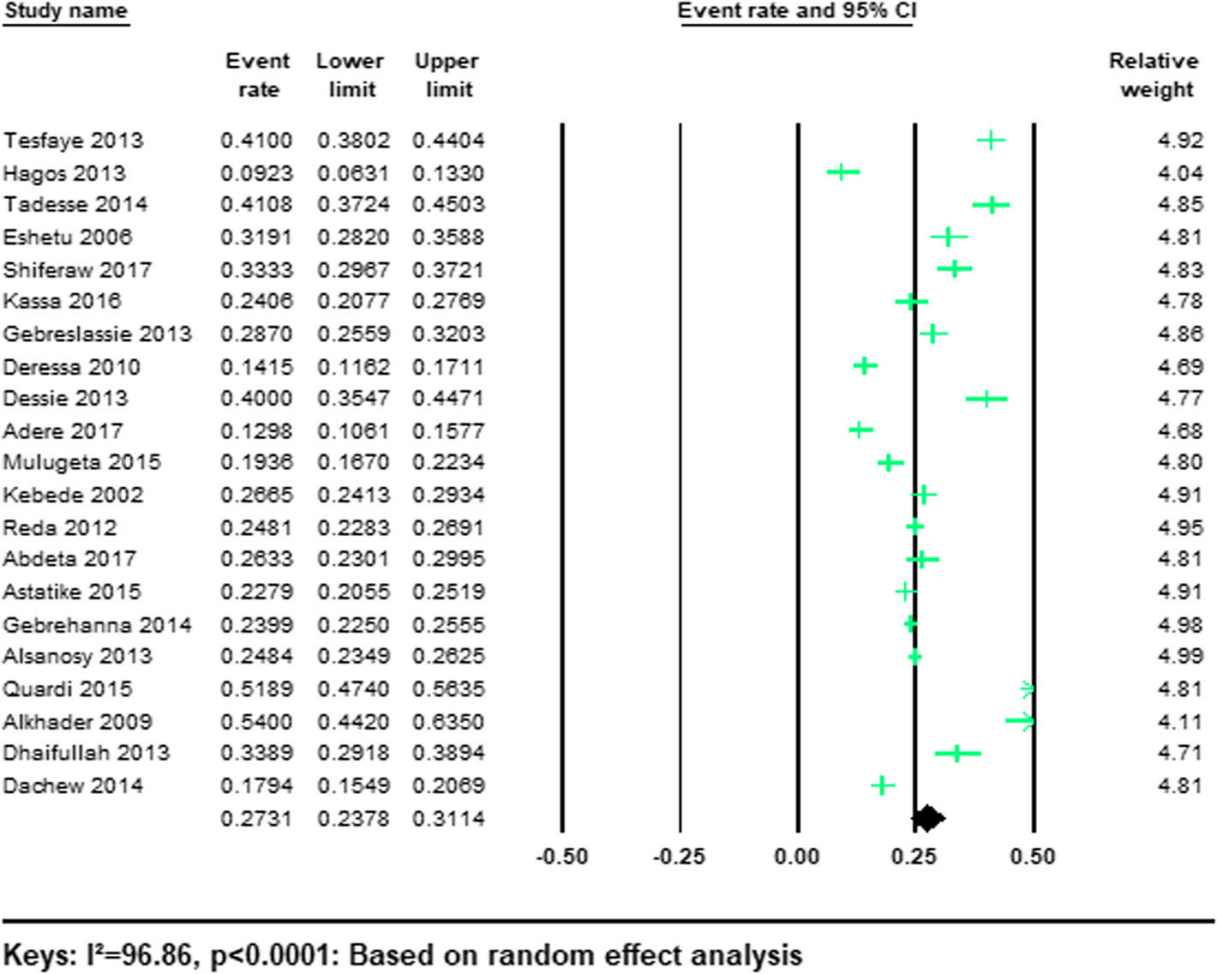
Forest plot of lifetime khat use among university students: a meta-analysis

As illustrated in Table 1, 21/25 (84%) studies reported the prevalence of lifetime khat use among university students. The total sample size of the participants from the included studies was 20,718. In our meta-analysis the pooled prevalence of lifetime khat use was 27.31% (95% CI; 23.78–31.14%) and we observed a significant heterogeneity (*I*^2^ = 96.86%; Q = 636.44, df = 20, *p* < 0.001). The forest plots of the prevalence of lifetime khat use were shown in Fig. 3.

The pooled prevalence of lifetime khat use was highest in Yemen (43.27, 95% CI 25.31–63.19%) and it was 24.82% (95% CI 21.30–28.72%) in Ethiopia and 37.32% (95% CI 15.74–65.48) in Saudi Arabia (Table 3). A few data were available, and the prevalence of current khat use among university students was very poorly recorded in countries where khat is consumed other than Ethiopia.

In our stratified analysis of included studies by gender we found that the lifetime pooled prevalence of khat use was higher in men 31.47% (95%CI 25.73–37.84) than in women 11.79%(95%CI 7.53–18.01). (See Table 3).

#### The risk of being male and current khat use

**Fig. 4:**
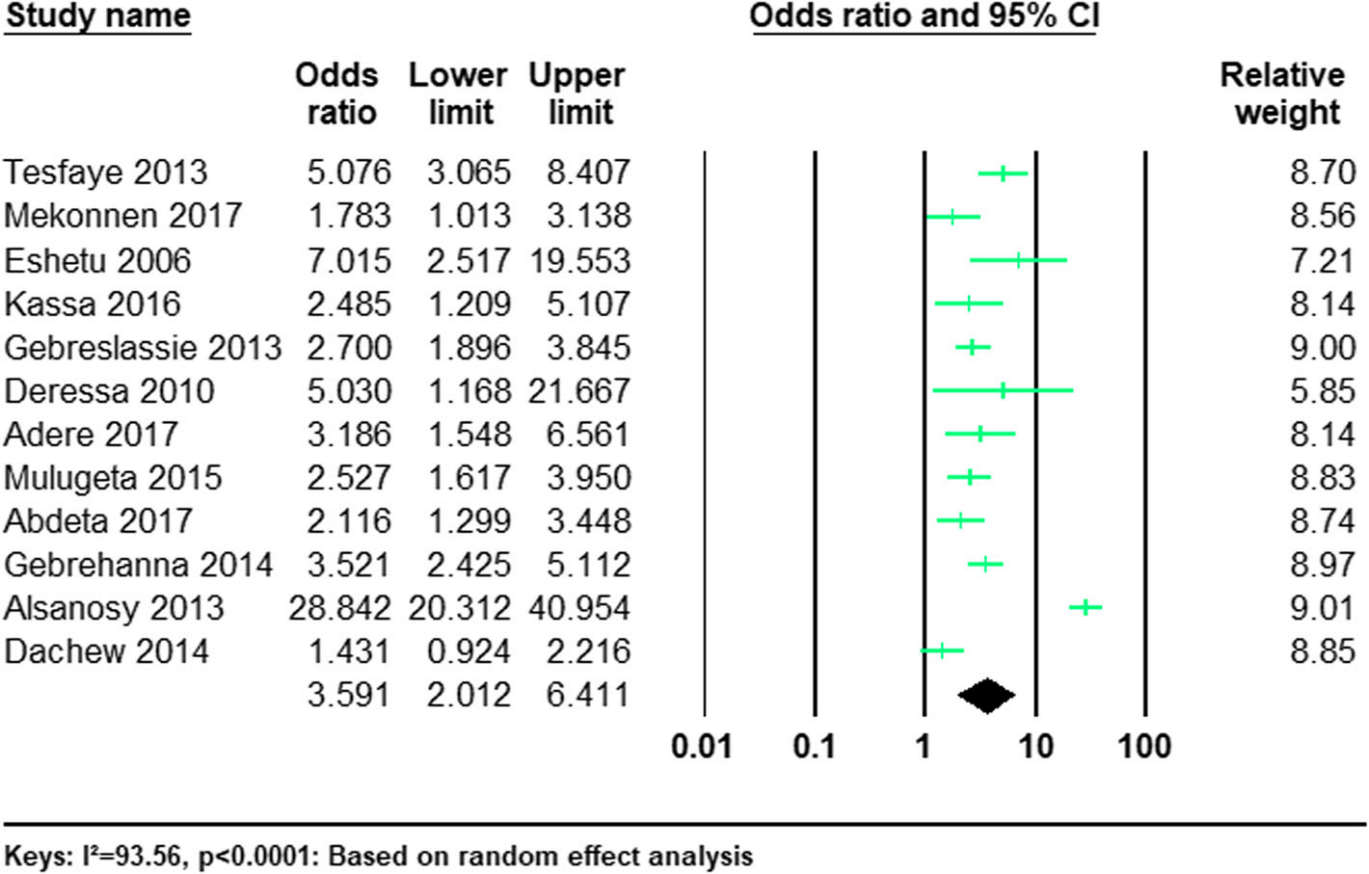
Forest plot of the odds of being male and current khat use among university students: a meta-analysis

Twelve of the studies provided information regarding the risk of current khat consumption in men and in women among university students (Table 1). The pooled odds ratio (OR) revealed that the odds of current khat use were significantly higher in men compared to women (OR 3.59; 95%CI 2.01–6.41, *P* < 0.0001). (See Fig. 4).

#### The risk of being male and lifetime khat use

**Fig. 5:**
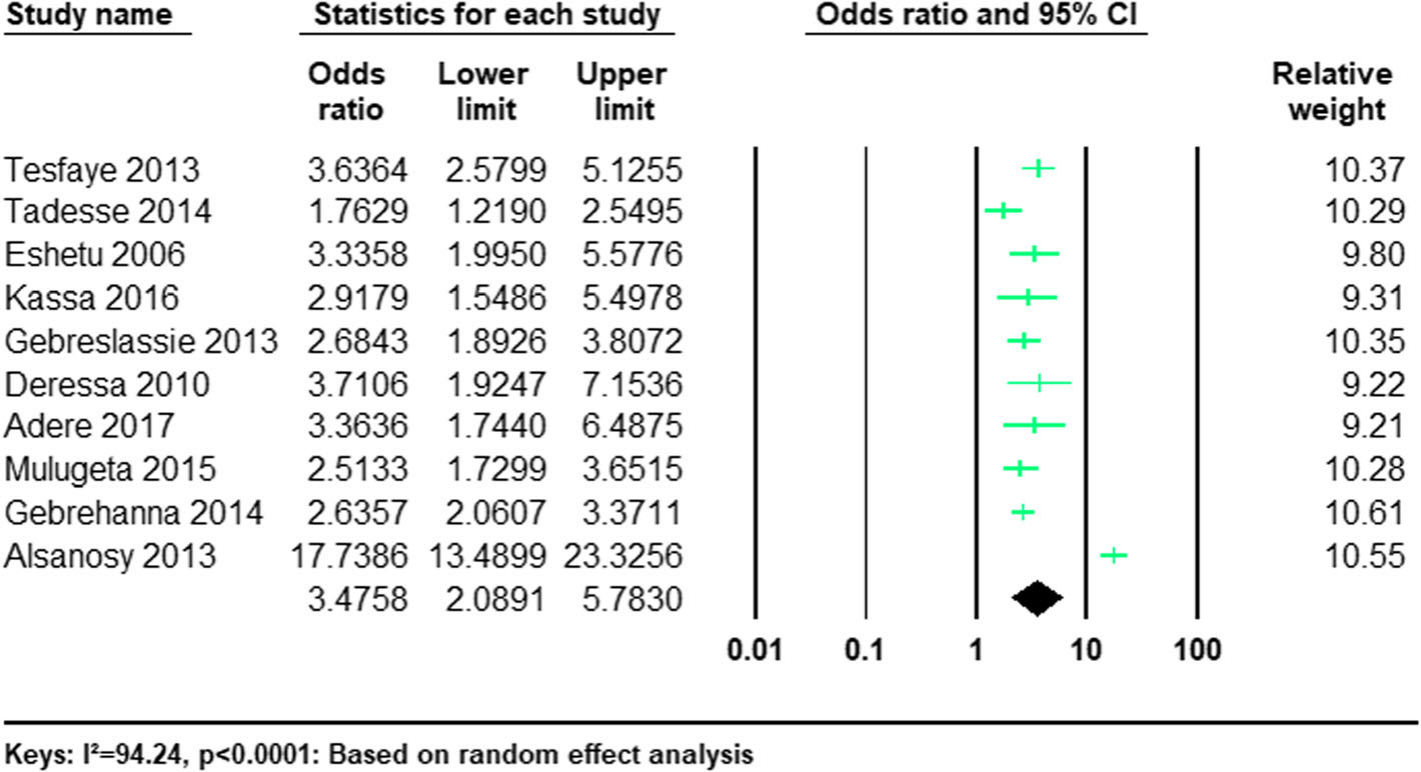
Forest plot of the odds of being male and lifetime khat use among university students: a meta-analysis

Ten of the studies provided information regarding the risk of lifetime khat use in men and in women university students (Table 1). The pooled odds ratio (OR) demonstrated that the odds of lifetime khat use were significantly higher in men compared with women (OR 3.48; 95%CI 2.09–5.78, *P* < 0.0001). (See Fig. 5).

#### Publication bias

**Fig. 6:**
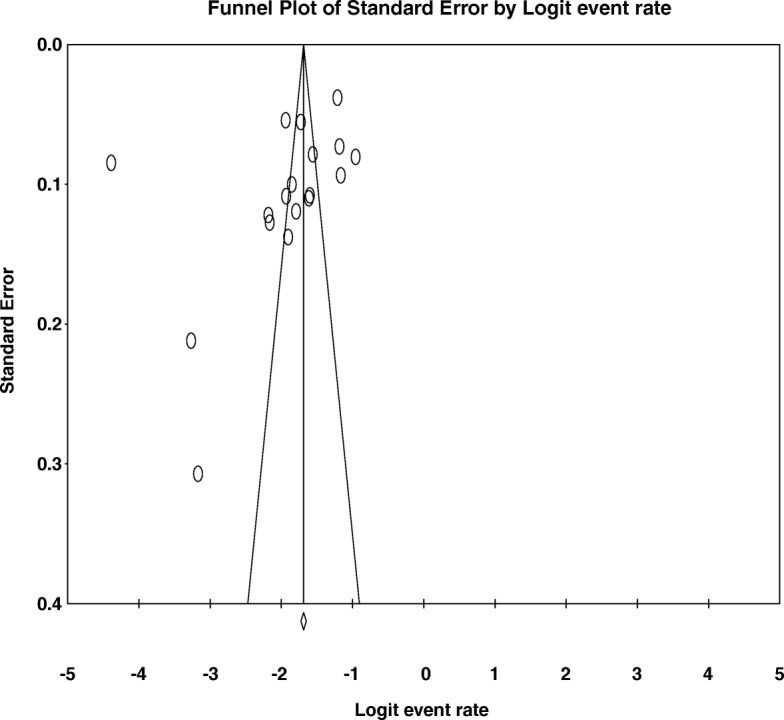
funnel plot of publication bias for current khat use among university

**Fig. 7:**
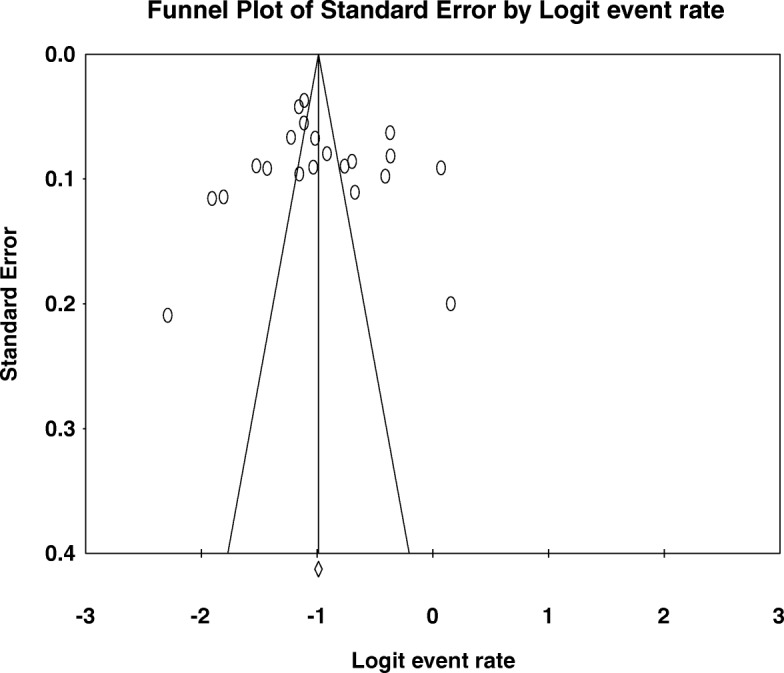
funnel plot of publication bias for lifetime Khat use among university

In our analysis of bias related for the effects of unincluded studies, as provided by funnel plot and Egger’s regression tests we found that there no evidence of substantial publication bias for the prevalence of current khat use ((B = − 7.75, SE = 4.95, *P* = 0.137) as well as lifetime khat use (B = 1.17, SE = 3.31, *P* = 0.727) among university student. (See Figs. 6 and 7).

#### Sensitivity analysis

For the purpose of further investigating the potential source of heterogeneity in the analysis of the prevalence of current and lifetime khat use among university students, we performed leave-one-out sensitivity analysis. In our sensitivity analysis, we found that our findings were robust and not dependent on a single study. Our pooled estimated prevalence varied between 11.73%(8.37–16.21%) and 14.38%(12.02–.17.88%) for the current and 26.28%(22.92–29.74%) and 28.41%(24.81–32.30%) for lifetime prevalence after deletion of a single study. (See Additional file 1 and Additional file 2).

## Discussion

To the best of our knowledge, this is the first meta-analysis that computed the current and lifetime prevalence of khat consumption among university students. In this meta-analysis, we investigated the prevalence of current and lifetime khat consumptions across 25 cross-sectional studies including a total of 24,136 participants. In addition, we evaluated the existing differences in the risk of khat consumptions between men and women in the last 30 days and consumption of khat in their lifetime among university students. Based on the results from our meta-analysis, the prevalence of both current and lifetime khat consumption was significantly higher in men than in women. We also found that males were 3.59 and 3.48 times more likely than females to have been users of khat in the last 30 days and in their lifetime respectively. Additionally, we found that a considerable proportion of students were users of khat in the last 30 days and in their lifetime. These findings indicate khat consumption is a significant health problem for university students as tobacco and alcohol consumptions [56–58].

In the present meta-analysis, in the included studies the evidence showed an apparent variation by study location (country) and the gender of the participants (male, female). Most of the included studies assessed both current and lifetime khat use and no study was found that determined khat dependence. A few data were available, and the prevalence of khat use among university students was very poorly recorded in countries where khat is commonly consumed other than Ethiopia.

In this meta-analysis, the pooled prevalence estimates of current khat use among university students were 14.16% (95% CI; 11.87–16.81). Whereas, the reported prevalence of current khat use ranges between 3.70% to 27.91% [41] depending on the study. The sociocultural and economic difference across the different countries where the studies were conducted maybe the main reasons for the observed variations in the current prevalence estimates of khat use among university students. In addition, the variability in the magnitude of khat use within the studied countries due to cultural difference as well as accessibility of khat in different areas within a single country might be the other reason for the difference in the observed magnitude. Our pooled prevalence estimate was in line with the general population study prevalence reports from 2011 Ethiopian demographic and health survey data 15.3% [59]. Our findings were lower than the general population prevalence estimates of the Jazan region of Saudi Arabia (28.7%%) [60] and occupational groups in Uganda (20.4%) [61]. The pooled prevalence of Khat chewing among university students is similar to cigarette smoking prevalence reports from meta-analysis which provided an overall estimation of smoking among students in Iran’s universities is 11.6%. However, the estimated prevalence of khat consumption among university students in our review is lower than a systematic review of a finding of the prevalence of alcohol use 31% among university students [62].

As expected, the estimated current khat consumption rate is higher for males than for females. This meta-analysis provided an estimate of a university student that consumption rates in the last 30 days of 19.26% for male university students and 6.41% for female university students. The possible reasons for this significant difference in magnitude of khat consumptions among male and female students may be due to the gender-related biological difference in the brain that possibly resulted in the observed variations in the magnitude of khat use between males and females [63]. The other possible difference might be sociocultural expectations and difference for these population groups. However, the exact reasons for the variation need further investigations. Similar with our finding the prevalence cigarette smoking is also higher in males than females as provided by systematic review evidence that resulted the frequency of smoking among the male and female students of Iran is 19.5 and 2.2%, respectively. The estimated prevalence of current khat use among male students our finding (19.26%) is similar to the finding of cigarette smoking among male university students (19.5%) but the estimate of khat consumption among females (6.41%) was higher than the estimates of cigarette smoking in female university students (2.2%).

In our stratified analysis included studies we identified that the pooled prevalence of current khat use among university students was highest in Saudi Arabia (18.85%) and it was 13.59% in Ethiopia and 13.04% in Yemen**.** This difference may be a sociocultural disparity between the countries. However, our results of the lifetime prevalence estimate khat consumption among university students were highest in Yemen (43.27%) and it was 24.82% in Ethiopia and 37.32% in Saudi Arabia. In our sensitivity, the difference among the studies across the varies countries was not statistically significant.

The results of the present meta-analysis show the pooled prevalence of lifetime khat consumption was 27.31% (95% CI; 23.78–31.14%). Our finding was in line with the general population study prevalence reports of khat use from the general population prevalence in of the Jazan region of Saudi Arabia (33.2%) [60]. However, the estimated prevalence of khat consumption among university students in our review is lower than the results of a systematic review of the prevalence of alcohol use 70% among young people [62].

As expected, the estimated lifetime khat consumption rate is higher for males than for females. This meta-analysis provided an estimate of university student lifetime khat consumption rates is 31.47% for male university students and 11.79% for female university students. Similar with our finding the prevalence cigarette smoking is also higher in males than females as provided by systematic review evidence that resulted in the frequency of ever alcohol use the male and female students is 53 and 50%, respectively. The estimated prevalence of lifetime khat use among male students our finding (31.47%) is lower than the finding of ever alcohol use in male university students (53%) [62]. Similarly, the estimate of lifetime khat consumption among females (11.79%) is lower than the estimates of alcohol use in female university students (50%) [62].

The results of the meta-analysis also demonstrated that gender was significantly associated with both current and lifetime khat consumptions among university students. Male students were 3.59 and 3.48 times more likely to have been current and lifetime khat users respectively than female students. This an increased risk of khat chewing in men might be due to the cultural norms of the countries. Moreover, gender-related variations in the brain might be the other possible reason for the observed difference [63].

### Strength and limitation of the study

The main strength of the present meta-analysis includes: First, we performed an extensive search of electronic databases and scanned references of the remaining articles to include all relevant literature to date; second, we used prespecified search strategy and data extraction was conducted by two independent investigators to minimize possible selection bias; third, a large number of studies were included in this study; fourth, we employed subgroup and sensitivity analysis to find publication bias and the risk of heterogeneity; fifth, we rigorously evaluated the quality of the included studies and the methodological quality of the included articles was generally good based on our assessment. However, we identified an apparent heterogeneity among the studies which we considered as limitations of the present meta-analysis. In addition, we observed that there is apparently a high variability in the magnitude of khat use within the studied countries and that a single prevalence rate per country hides this variance. Finally, since the studies included in this meta-analysis were conducted only in three countries (Ethiopia, Saudi Arabia, Yemen) the findings may not be generalizable to the other population.

## Conclusion

The results of the present meta-analysis show the pooled prevalence of current and lifetime khat consumption among university students were 14.16 and 27.31%, respectively. In our stratified analysis, both the current and lifetime prevalence of khat use were higher in men than in women. We also found that males were 3.59 and 3.48 times more likely than females to be users of khat in the last 30 days and their lifetime respectively.

Even though khat use is banned in Saudi Arabia, both the current and lifetime prevalence estimates of khat consumption among university students were common and comparable with the magnitude in Ethiopia and in Yemen.

Programmes that specifically aimed at increasing awareness and that most motivate reduced khat consumption among university students were recommended. In addition, preventions and interventions programs on khat consumption among university students should be tailored depending on the gender and existing sociocultural and environmental considerations. Future epidemiological studies focusing on the reasons for the observed gender difference in the magnitude of khat consumptions could be conducted. Furthermore, future longitudinal studies focused on incidence and risk factors of khat consumption among university students and studies on better ways to reduce khat consumptions among university students are recommended.

## Additional files

Additional file 1: Sensitivity analysis of prevalence for each study being removed at a time: prevalence and 95% confidence interval of current Khat use among university. (DOCX 18 kb)

Additional file 2: Sensitivity analysis of prevalence for each study being removed at a time: prevalence and 95% confidence interval of Khat use among university. (DOCX 18 kb)

## Abbreviations

CI: Confidence Interval
EMBASE: Excerpta Medica Database
OR: Odds Ratio
PRISMA: Preferred Reporting Items for Systematic Reviews and Meta-Analyses
