# Supplementary material for: Epidemiology of khat (Catha edulis) consumption among university students: a meta-analysis
Source: BMC Public Health. 2019 Feb 4;19:150. doi: 10.1186/s12889-019-6495-9 (PMC6360776; doi:10.1186/s12889-019-6495-9)
Supplement: Supplementary file 1 — Sensitivity analysis of prevalence for each study being removed at a time: prevalence and 95% confidence interval of current Khat use among university. (DOCX 18 kb) [file 12889_2019_6495_MOESM1_ESM.docx]

**Additional file 1:** Sensitivity analysis of prevalence for each study being removed at a time: prevalence and 95% confidence interval of current khat use among university students

| Study excluded | prevalence | 95%CI |
| --- | --- | --- |
| Dida N. et.al (2014) [33] | 12.13 | 8.62-16.81 |
| Tesfaye G. et. al (2013) (34) | 11.87 | 8.41-16.49 |
| Hagos EG. et. al (2013) (35) | 13.07 | 9.40-17.88 |
| Mekonnen T. et.al (2017) [36) | 12.51 | 8.93-17.25 |
| Eshetu E.et. al (2006) (38) | 12.26 | 8.72-16.96 |
| Kassa A.et. al (2016) (40) | 12.14 | 8.63-16.82 |
| Gebreslassie M. et.al (2013) (41) | 11.73 | 8.37-16.21 |
| Deressa W. et.al (2010) (45) | 13.19 | 9.51-18.00 |
| Adere A. et.al (2017) (44) | 12.50 | 8.92-17.24 |
| Mulugeta Y. et.al (2015) (45) | 12.35 | 8.78-17.08 |
| Kebede Y (2002) (46) | 12.10 | 8.55-16.86 |
| Abdeta et al. (2017) (47) | 11.86 | 8.43-16.43 |
| Astatkie et al. (2015) (48) | 14.38 | 12.02-17.12 |
| Gebrehanna et al. (2014) (49) | 12.34 | 8.63-17.34 |
| Alsanosy et al. (2013) (51) | 11.88 | 8.33-16.55 |
| Ageelyet.al. (2009) (27) | 12.20 | 8.51-17.18 |
| Kubas Ma et.al. 2015 [53]   \|  \| \| --- \| | 12.33 | 8.79-17.03 |
| Dachew BA.et. al (2014) (50) | 12.29 | 8.73-17.04 |

Key. The analysis is based on random effect model
