# Supplementary material for: Epidemiology of khat (Catha edulis) consumption among university students: a meta-analysis
Source: BMC Public Health. 2019 Feb 4;19:150. doi: 10.1186/s12889-019-6495-9 (PMC6360776; doi:10.1186/s12889-019-6495-9)
Supplement: Supplementary file 2 — Sensitivity analysis of prevalence for each study being removed at a time: prevalence and 95% confidence interval of Khat use among university. (DOCX 18 kb) [file 12889_2019_6495_MOESM2_ESM.docx]

**Additional file 2:** Sensitivity analysis of prevalence for each study being removed at a time: prevalence and 95% confidence interval of lifetime khat use among university students

| Study excluded | prevalence | 95%CI |
| --- | --- | --- |
| Dida N. et.al (2014) [36] | 26.68 | 23.29-30.38 |
| HagosEG. et. al (2013) (35) | 28.41 | 24.81-32.30 |
| Tadesse M. et.el.(2014) (37) | 26.69 | 23.22-30.47 |
| Eshetu E.et. al (2006) (38) | 27.08 | 23.44-31.06 |
| Shiferaw D.et. al (2017) (39) | 27.02 | 23.39-30.98 |
| Kassa A.et. al (2016) (40) | 27.47 | 23.80-31.48 |
| Gebreslassie M. et.al (2013) (41) | 27.23 | 23.55-31.26 |
| Deressa W. et.al (2010) (42) | 28.12 | 24.54-32.00 |
| Dessie y. et.al (2013) (43) | 26.74 | 23.22-30.58 |
| Adere A. et.al (2017) (44) | 28.22 | 24.67-32.07 |
| Mulugeta Y. et.al (2015) (45) | 27.77 | 24.11-31.73 |
| Kebede et.al. (2002) (46) | 27.34 | 23.61-31.41 |
| Reda AA et.al (2012) [25] | 27.43 | 23.66-31.57 |
| Abdeta et al. (2017) (47) | 27.35 | 23.67-31.37 |
| Astatkie et al. (2015) (48) | 27.55 | 23.83-31.61 |
| Gebrehanna et al. (2014) (49) | 27.48 | 23.62-31.70 |
| Alsanosy et al. (2013) (51) | 27.43 | 23.50-31.74 |
| Quadri et al. (2015) (52) | 26.28 | 23.08-29.74 |
| Alkhader nl et.al (2009) (54) | 26.36 | 22.92-30.11 |
| Dhaifullah et.al (2013) (55) | 27.00 | 23.40-30.94 |
| Dachew BA.et. al (2014) (50) | 27.85 | 24.23-31.80 |

Key. The analysis is based on random effect model
